# Dual protective role of velutin against articular cartilage degeneration and subchondral bone loss *via* the p38 signaling pathway in murine osteoarthritis

**DOI:** 10.3389/fendo.2022.926934

**Published:** 2022-07-22

**Authors:** Kelei Wang, Xuanyuan Lu, Xinyu Li, Yufeng Zhang, Rongjian Xu, Yun Lou, Yanben Wang, Tan Zhang, Yu Qian

**Affiliations:** ^1^ Department of Orthopedics, Shaoxing People’s Hospital (Shaoxing Hospital, Zhejiang University School of Medicine), Shaoxing, China; ^2^ Department of Orthopedics, Sir Run Run Shaw Hospital, School of Medicine, Zhejiang University, Hangzhou, China; ^3^ Department of Orthopedics, The Second Affiliated Hospital and Yuying Children’s Hospital of Wenzhou Medical University, Wenzhou, China

**Keywords:** velutin, chondrocyte, subchondral bone, p38 pathway, osteoarthritis

## Abstract

Osteoarthritis (OA) is a common degenerative joint condition associated with inflammation and characterized by progressive degradation of the articular cartilage and subchondral bone loss in the early stages. Inflammation is closely associated with these two major pathophysiological changes in OA. Velutin, a flavonoid family member, reportedly exerts anti-inflammatory effects. However, the therapeutic effects of velutin in OA have not yet been characterized. In this study, we explore the effects of velutin in an OA mouse model. Histological staining and micro-CT revealed that velutin had a protective effect against cartilage degradation and subchondral bone loss in an OA mouse model generated by surgical destabilization of the medial meniscus (DMM). Additionally, velutin rescued IL-1β-induced inflammation in chondrocytes and inhibited RANKL-induced osteoclast formation and bone resorption *in vitro*. Mechanistically, the p38 signaling pathway was found to be implicated in the inhibitory effects of velutin. Our study reveals the dual protective effects of velutin against cartilage degradation and subchondral bone loss by inhibiting the p38 signaling pathway, thereby highlighting velutin as an alternative treatment for OA.

## Introduction

Osteoarthritis (OA) is the most common degenerative joint condition. The incidence of OA is increasing, especially in the elderly. Chronic OA causes pain and disability (1, 2) and may also negatively impact the mental health of affected individuals, possibly causing the development of depressive symptoms and perceived memory loss (3, 4).

Owing to the increasing understanding of OA pathology, it is now accepted that due to various reasons, all joint structures are impacted during the course of OA and that degradation or loss of articular cartilage is a prominent feature of OA development (5). During OA progression, chondrocytes remodel the extracellular matrix (ECM) of articular cartilage (6). Moreover, inflammation is involved in the development of OA even in the early stages of the condition, and inflammatory cytokines such as TNF-α and IL-1β are recognized as the most important compounds in OA progression (7, 8). The p38 signaling pathway is a classic inflammatory pathway that exerts a crucial role in the course of a variety of human diseases, including OA (9–11). Inhibiting the P38 signaling pathway reduces the release of downstream inflammatory factors and inhibits chondrocyte apoptosis, which may contribute to cartilage degradation (12), suggesting that protection against articular cartilage degeneration is a possible therapeutic target in OA (13).

Furthermore, subchondral bone also plays a pivotal role in the evolution of OA except for articular cartilage. The release of inflammatory and osteoclastic stimulating factors from the articular cartilage may contribute to the deterioration of the subchondral bone during OA progression (14–16). In the early stages of OA, reduced mechanical loading of articular cartilage increases nuclear factor-κB receptor activator ligand (RANKL) expression in osteocytes, causing excessive osteoclastogenesis and high bone resorption activity, ultimately leading to subchondral bone loss (17, 18). The release of inflammatory factors also promotes osteoclast formation and bone resorption, leading to subchondral bone loss (19, 20). RANKL can activate the P38 signaling pathway that, in turn, activates transcription factors, such as c-Fos and NFATc1, both of which are crucial for osteoclast development and function. Thus, inhibition of the p38 signaling pathway can prevent the formation of osteoclasts and protect against bone loss in the subchondral bone (21, 22). These considerations provide compelling evidence for treating OA in its early stages by targeting the bone resorption activity of osteoclasts (23).

Current treatments of OA, including pharmacological and nonpharmacological treatments, target either cartilage degradation or subchondral bone loss. Due to the uncertainty and randomness of nonpharmacological treatments, pharmaceutical drugs remain the primary treatment option for OA (24). Although common drugs currently used to treat OA, such as non-steroidal anti-inflammatory drugs (NSAIDs), can be remarkably effective in controlling symptoms, they are associated with complications such as cardiovascular and gastrointestinal side effects (25, 26). As a result, novel therapeutic measures for OA are urgently needed, preferably with dual protective effects against articular cartilage degradation and bone loss of the subchondral bone during OA development.

Velutin, a glycoside extracted from mistletoe, exerts protective effects against a variety of diseases and effectively inhibits the expression of the pro-inflammatory cytokines, including TNF-α and IL-1β, by inhibiting NF-κB and p38 activation (27). Although the anti-inflammatory properties of velutin have long been shown in several studies, indicating its potential in reducing inflammatory bone loss (28, 29), there is still little known about its role in OA.

Therefore, in this study, we investigate whether velutin exerts a protective effect against cartilage degeneration and SCB loss during OA progression. Our results reveal a dual protective effect in mice OA models, which is reflected by AC degeneration and SCB loss. Additionally, velutin suppressed chondrocyte inflammation and prevented the development of osteoclasts and bone resorption *in vitro*. Notably, the dual protective effects of velutin were mainly mediated *via* the p38 signaling pathway. In summary, our findings demonstrate the dual therapeutic effect of velutin in OA.

## Materials and methods

### Reagents

Velutin was purchased from ChemFace (Wuhan, Hubei, China). Primary antibodies against CollagenII (ab34712), CollagenX (ab58632), ADAMTS5 (ab41037), Actin (ab8227) and the secondary fluorescence antibodies (ab150077) were obtained from Abcam (Cambridge, UK). Primary antibodies against Cox2 (#12282), c-Fos (#2250), NFATc1(#8032), p-p38 (#4511), p38 (#8690), p-ERK (#4370), ERK (#4695) p-JNK (#4668), JNK (#9258), p-p65 (#3033), p65 (#8242) and the secondary antibody (#7074) were obtained from Cell Signaling Technology (Danvers, MA, USA). Primary antibody against MMP3 (NBP2-75931) was bought from NOVUS Biologicals (Colorado, America). PeproTech (Rocky Hill, NJ, USA) provided the recombinant mouse IL-1β. Gibco (Rockville, MD, USA) provided Alpha-modified minimal essential medium (α-MEM), Dulbecco’s modified Eagle medium (DMEM/F12), fetal bovine serum (FBS), Trypsin-EDTA (0.25%), penicillin, and streptomycin. RANKL and M-CSF were obtained from R&D Systems (Minneapolis, MN, USA). The type II collagenase was given by Sigma-Aldrich (St. Louis, MO, USA). SolarBio provided Safranin O and Fast Green, as well as hematoxylin and eosin solution (Beijing, China). Beyotime Institute of Biotechnology supplied the Cell Counting Kit-8 (CCK8) and BCA (Shanghai, China).

### Animal experiments

All animal experimental procedures, animal husbandry, and care were received permission from the Animal Ethical Committee of Shaoxing People’s Hospital, Shaoxing, Zhejiang Province, China (No. 82072467). Thirty 7-week-old C57BL/6 male wild type mice were used in this study and were housed in specialized cages at 24–26°C. Surgical destabilization of the medial meniscus (DMM) was utilized to create OA models in mice. All mice were divided into three groups at random (10 mice per group): the sham (Sham), DMM [DMM+1% dimethyl sulfoxide (DMSO) treatment], and velutin-treated groups (DMM+velutin). Pentobarbital (35 mg/kg) was used to anesthetize mice. The modified DMM surgery for mice OA in this study was conducted as previously reported (30–33). We carefully resected the medial meniscus of the knee joint, then sutured the skin and injected antibiotics for three days at the heels of the surgery. Seven days following DMM surgery, the velutin-treated group was treated with velutin (32 mM) through joint injection with micro syringe. Velutin injection was administered once a week at a concentration of 32uM for 5ul each time for eight weeks until mice were sacrificed. Mice in the DMM group were treated with 1% DMSO by joint injection. After eight weeks, the procedure was completed; mice were sacrificed, and tissue samples were collected from the knee joints for subsequent histological analysis.

### Micro-CT scanning and histological analysis

On the day of sacrifice, knee tissue samples of mice were gathered from the above three groups and fixed above tissue samples in 4% paraformaldehyde for 48 h before scanning using a μCT100 high-resolution cabinet cone-beam micro-CT scanner (Scanco Medical Wangen-Brüttisellen, Switzerland). At an X-ray voltage of 70 kV, a current of 200 A, and an isometric resolution of 20 m, serial tomographic pictures were recorded. After decalcifying with 10% EDTA for three weeks and changing the 10% EDTA daily until decalcification was complete, all samples were subjected to dehydration, embedding, and coronal sectioning. Then, using Safranin O and hematoxylin and eosin (H&E) staining, cartilage degradation and subchondral bone loss were observed. The Osteoarthritis Research Society International (OARSI) standards were used to score cartilage deterioration (34). TRAP staining was performed to measure damage to the subchondral bone. Immunohistochemical analysis was carried out to evaluate p-p38 MAPK expression (purchased from Cell Signaling Technology, Danvers, MA, USA).

### Parameter measurement

The area between the superficial surface of articular cartilage and the most superficial tidemark was defined as the articular cartilage (AC) area; the area between the most superficial tidemark and the calcified cartilage-subchondral bone junction was defined as the calcified cartilage (CC) area; the area between the calcified cartilage-subchondral bone junction and the most superficial boundary of the marrow spaces was defined as the subchondral bone (SCB) area. Osteophyte score: quantification by osteophyte maturity from 1 to 3 (0 = none, 1 = mostly cartilaginous, 2 = mixed cartilage and bone with active vascular invasion and endochondral ossification, 3 = mostly bone) (35–37).

### Cell culture and concentrations of reagents

The chondrocytes were extracted from the knee joints of 2-day-old C57BL/6 mice and cultured in complete DMEM/F12 medium with 10% FBS, 100 U/mL penicillin, 100 mg/mL streptomycin, and 0.1% type II collagenase, and then incubated for 8 h at 37°C with 5% CO_2_. After enzymatic digestion, cartilage tissues were collected, the medium was replaced by a medium without type II collagenase, and cells were used in subsequent experiments. Bone marrow-derived macrophages (BMMs) were isolated from the hind limb bone marrow cavity of 5–7-week-old C57BL/6 mice and cultured in complete α-MEM medium with 10% FBS, 100 U/mL penicillin, 100 mg/mL streptomycin in an incubator at 37°C with 5% CO_2_ for 24 h. The following day, the medium was added with M-CSF and RANKL in the absence or presence of velutin (1, 2, 4 μM). To test time-dependent effects, we used the maximum concentration of velutin (4 μM). The medium was changed every two days. In the end, the cells were fixed with 4% paraformaldehyde (PFA) for 20 minutes at room temperature, followed by washing with PBS for three times and staining with TRAP. Cells with more than 3 nuclei were identified as osteoclasts. In tis study, the concentrations of various reagents used were as follows: IL-1β-10 ng/ml; M-CSF-25 ng/ml; RANKL-100 ng/mL.

### High-cell density culture of primary mouse chondrocytes

</u>Complete DMEM/F12 medium was used to grow chondrocytes. Chondrocytes were extracted using 0.25% trypsin-EDTA (Gibco, Thermo Fisher Scientific, Waltham, MA, USA) once the cells had reached 80–90% confluency, and cells were transplanted into 12-well plates at a density of 3 × 10^5^ cells/well. After overnight incubation, the medium was changed [supplemented with IL-1β only or IL-1β and velutin (32 μM, according to the CCK8 result)]. For the control group, chondrocytes were cultured without IL-1β or velutin. The media was replaced daily, and the cells were fixed in 4% paraformaldehyde for 30 min after 7 days of incubation. After this process, we stained with Safranin O to estimate the protective effect of velutin against IL-1β-induced chondrocyte inflammation.

### Culture of human cartilage tissue

All human cartilage tissue experimental procedures were approved by Medical Ethics Committee of Shaoxing People’s Hospital, Shaoxing, Zhejiang Province, China (No.2019-K-064-01) and informed consent from the patient was obtained. Human cartilage tissue was collected from an OA patient hospitalized for total joint replacement surgery. Cartilages were excised from the tibial plateau during total knee replacement surgery and cultured in DMEM/high-glucose medium and incubated at 37°C with 5% CO_2_ for 24 h with 10% FBS and 1% antibiotic/antimycotic solution (100 U/mL penicillin and 100 mg/mL streptomycin) (38). The treatment medium was subsequently added [supplemented with IL-1β only or IL-1β and velutin (32 μM)]. The tissue was cultured in the treatment medium for 7 days. For the control group, tissue was cultured in DMEM/high-glucose with no IL-1β or velutin. The medium was changed daily; 7 days later, the human cartilage tissue was collected and fixed in 4% paraformaldehyde, decalcified and paraffin embedded for histological analysis.

### Cytotoxicity assays

Chondrocytes were seeded at a density of 8 × 10^3^ cells/well in 96-well plates and cultured for 24 or 48 h in a medium containing varying doses of velutin (0, 1,2, 4, 8, 16, 32, 64, and 128 M). The concentration of each velutin was measured in five wells. Next, each well was added 10 μL of CCK8 solution at the appropriate time periods, and chondrocytes were cultured for 1 h. A spectrophotometer was used to measure absorbance at 450 nm at the conclusion of the experiment (Thermo Scientific, Multiskan GO, Waltham, MA, USA).

### Western blotting

Chondrocytes were seeded at a density of 2 × 10^5^ cells per well in 6-well plates in DMEM/F-12 for 24 h. Cells were subsequently stimulated with IL-1β alone or IL-1β with velutin for an additional 24 h after being pretreated with velutin for 2 h.

BMMs were seeded at a density of 5 × 10^5^ cells/well in α-MEM for 24 h in 6-well plates. Prior to receiving RANKL stimulation for 0, 5, 15, 30, and 60 min, BMMs were pretreated with or without 4 μM velutin for 2 h. In addition, BMMs were stimulated with RANKL for 1, 3, or 5 days, with or without 4 μM velutin. Total proteins were collected using RIPA lysis buffer, followed by a 20-minute incubation period on ice before being spun for 15 min at 13,300 rpm at 4°C. We used bicinchoninic acid (BCA) for protein quantification and mixed the proteins with sodium dodecyl sulfate sampling buffer, and then incubated at 95°C for 10 min. After electrophoresis, proteins from each group were transferred to PVDF membranes (Bio-Rad Laboratories Inc., Hercules, CA, USA). Specific primary antibodies were added after blocking with 5% nonfat milk for 1 h at 20–25°C, and samples were incubated overnight at 4°C. Then, the samples were treated for 1 h at 20–25°C with secondary antibodies. At the end of the experiment, we used enhanced chemiluminescence (ECL) solution to detect fluorescent signals.

### qRT-PCR

Total RNA was isolated from chondrocytes using the TRIzol reagent (Thermo Fisher Scientific, Waltham, MA, USA). Chondrocytes and BMMs were seeded at a density of 2 × 10^5^ cells per well in 6-well plates and incubated for 24 h. After that, chondrocytes were stimulated with IL-1β at various concentrations (4, 8, 16, and 32 μM) of velutin for 24 h. BMMs were stimulated with RANKL and various concentrations of velutin (1, 2, and 4 μM) for 6 days until the osteoclast formed. Single-stranded complementary DNA was synthesized using the PrimeScript RT Master Mix (Takara Bio Inc., Kusatsu, Shiga, Japan). On the ABI StepOnePlus System, PCR was done in triplicate with 1 µL of cDNA as a template using Power SYBR^®^ Green PCR Master Mix (TakaraBio Inc.) and the Power SYBR^®^ Green PCR Master Mix (TakaraBio Inc.; Applied Biosystems, Warrington, UK). The expression of the target gene was compared to that of β-actin. Primer information is shown in **Table 1**.

**Table 1:** Primer sequences for RT-PCR.

Gene	Forward primer (5’to3’)	Reverse primer (5’to3’)
β-actin	AGCCATGTACGTAGCCATCC	CTCTCAGCAGTGGTGGTGAA
TNF-α	CAGGCGGTGCCTATGTCTC	CGATCACCCCGAAGTTCAGTAG
IL-6	TAGTCCTTCCTACCCCAATTTCC	TTGGTCCTTAGCCACTCCTTC
CollagenII	CAGGATGCCCGAAAATTAGGG	ACCACGATCACCTCTGGGT
CollagenX	TTCTGCTGCTAATGTTCTTGACC	GGGATGAAGTATTGTGTCTTGGG
MMP3	ACATGGAGACTTTGTCCCTTTTG	TTGGCTGAGTGGTAGAGTCCC
ADMTS5	GGAGCGAGGCCATTTACAAC	CGTAGACAAGGTAGCCCACTTT
COX2	TGAGCAACTATTCCAAACCAGC	GCACGTAGTCTTCGATCACTATC
CTSK	CTCGGCGTTTAATTTGGGAGA	TCGAGAGGGAGGTATTCTGAGT
TRAP	CACTCCCACCCTGAGATTTGT	CCCCAGAGACATGATGAAGTCA
V-ATPase d2	CTGGTTCGAGGATGCAAAGC	GTTGCCATAGTCCGTGGTCTG
NFATc1	CAGTGTGACCGAAGATACCTGG	TCGAGACTTGATAGGGACCCC
c-Fos	CGGGTTTCAACGCCGACTA	TGGCACTAGAGACGGACAGAT
DC-STAMP	CTGTGTCCTCCCGCTGAATAA	AGCCGATACAGCAGATAGTCC

### Osteoclast differentiation assay

BMMs were planted at a density of 8 × 10^3^ cells/well for 24 h in a full α-MEM medium containing M-CSF. Then, cells were stimulated with RANKL and various doses (1, 2, or 4 μmol/L) of velutin for 6 days until osteoclast formed in the RANKL-stimulated wells. Next, to explore the time dependence of velutin on osteoclast differentiation, we stimulated osteoclasts with the most inhibitory velutin concentration (4 μmol/L, as determined in the previous step) at three time periods (D0–D2 defined as early-stage; D2–D4 defined as middle stage; D4–D6 defined as late-stage) until the presence of osteoclasts with more than three nuclei in the control group (treated with RANKL only) was observed, approximately 6 days. After that, cells were fixed and TRAP staining was used to stain the cells. Mature osteoclasts were defined as Trap (+) cells with more than three nuclei.

### Bone resorption assays

In a complete α‐MEM medium, BMMs were seeded onto bovine cortical bone slices in 96-well culture plates with or without various concentrations of velutin (1, 2, or 4 μM). This experimental procedure was performed in the presence of RANKL, and the density of osteoclasts was 8 × 10*^3^* cells/well. Media was changed every other day until osteoclasts and absorption pits were formed before bone slices were collected. This process usually lasts 15 days. With the help of a scanning electron microscope (FEI Quanta 250), the area of the resorption pits was measured using ImageJ software after they were analyzed.

### Immunofluorescence microscopy

To explore the immunofluorescence of p-p38 MAPK, chondrocytes were seeded in a 12-well plate at a density of 10 × 10^4^ cells/well with glass coverslips. After overnight incubation, the medium of the IL-1β group (supplemented with IL-1β) and the velutin treatment group was replaced. For the control group, chondrocytes were cultured without IL-1β or velutin. After rinsing the glass coverslips four times in PBS at the conclusion of the incubation period, cells were fixed for 15 min and Triton X-100 was used to permeabilize the cells for 10 min at 20–25°C before blocking with BSA for 2 h. Then, cells were treated overnight at 4°C with primary antibody against p-p38 (#9211, CST, 1:400), followed by washing with PBS four times. Finally, cells were incubated with the secondary antibody for 1 h at 20–25°C and stained with DAPI for 5 min. Images were taken with an Olympus FV1200 microscope (Olympus Corporation, Shinjuku, Tokyo, Japan) and a Nikon ECLIPSE Ti microscope (Nikon Instruments Inc., Minato, Tokyo, Japan). ImageJ software was used to calculate the fluorescence intensity.

### Statistical analysis

Each experiment was repeated at least three times in total. The data are presented as means and standard deviations (SD). For statistical analysis, Student’s t-tests and one-way ANOVA were utilized. Statistical significance was set at *P* < 0.05.

## Results

### Velutin treatment has a dual protective effect in a DMM-induced OA mouse model

**Figure 1:**
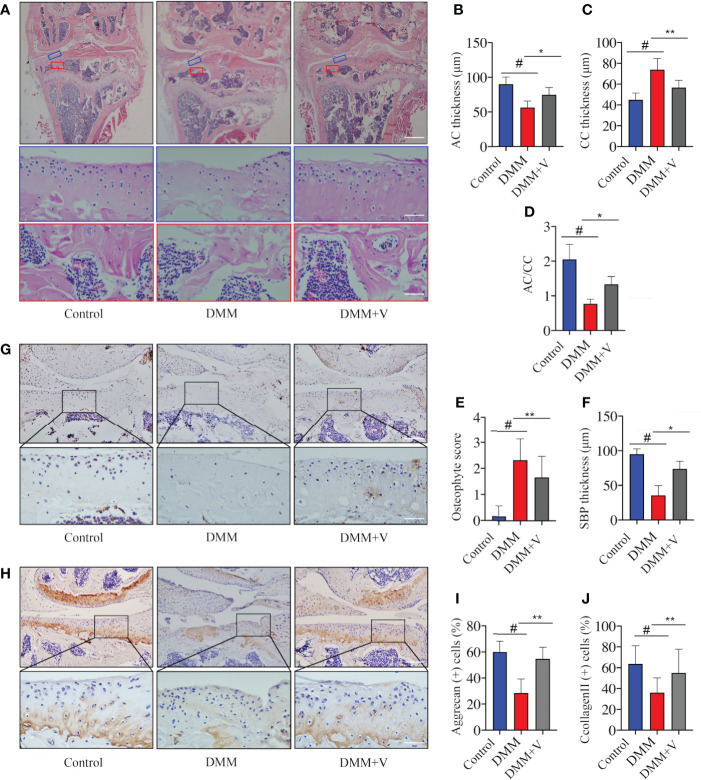
Velutin reduces osteoarthritis (OA) progression in a DMM-induced OA mouse model *in vivo*. **(A)** Hematoxylin and eosin staining in different experimental groups (control, DMM, and DMM+velutin groups). The blue rectangle represents the local magnification of the articular cartilage and the red rectangle represents the local magnification of the subchondral bone. **(B–F)** Statistics of each parameter of articular cartilage and subchondral bone. **(G–J)** Immunohistochemical (IHC) staining of aggrecan **(G)** and type II Collagen **(H)** in different experimental groups (control, DMM, and DMM+velutin groups). Scale bar: 100 μm; n = 6 per group. Data are presented as mean ± SD; ^#^ *P* < 0.01 vs. control group; *P < 0.05, **P < 0.01.

To investigate the effects of velutin in a DMM-induced OA mouse model, histological staining was used. As shown in **Figure 1A**, the knee joints from each group were imaged after H&E staining. Compared to the DMM group, the velutin-treated group exhibited noticeably more surface regularity, a thinner CC layer, a thicker AC layer, and a lower osteophyte score. Correlative histological analysis revealed a protective effect of velutin against cartilage degeneration during OA progression. Additionally, compared to the DMM group, the SCB in the velutin-treated group was much thicker, suggesting a protective effect against SCB loss (**Figures 1A** **–F**). Immunohistochemical analysis revealed that Aggrecan and collagen II expression were much lower in the DMM group, but were reversed in the velutin treatment group (**Figures 1H–J**). This suggests that velutin exhibited a dual protective effect in DMM-induced OA mice by protecting against AC degradation and rescuing the SCB loss in the early stages of osteoarthritis.

### Velutin treatment inhibits articular cartilage degradation and SCB loss in a DMM-induced OA mouse model

**Figure 2:**
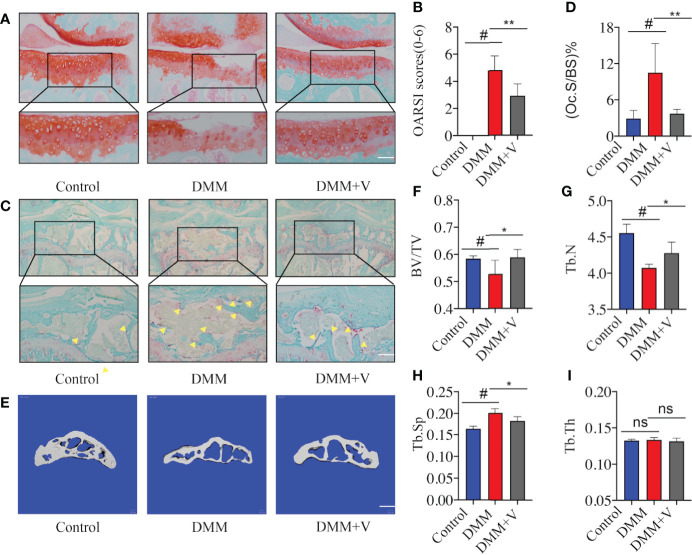
Velutin treatment reduces articular cartilage degeneration and subchondral bone deterioration in mice with DMM-induced OA. **(A)** Representative Safranin O staining of articular cartilage in distinct experimental groups; scale bar: 100 μm. **(B)** Osteoarthritis Research Society International (OARSI) scores of the cartilage. **(C, D)** Representative photos of fast green-stained subchondral bones and TRAP-stained osteoclasts in subchondral bone of the knee joint; the yellow triangle represents osteoclasts; scale bar: 100 μm. **(E-I)** Three-dimensional micro-CT images of the medial compartment of the tibial subchondral bone in sagittal views (control, DMM, and DMM+velutin groups); scale bar: 100 μm. Three-dimensional structural characteristics of the tibial subchondral bone histogram: (BV/TV) trabecular bone volume/tissue volume, (Tb.N) trabecular number, (Tb. Th) trabecular thickness, (Tb.Sp) trabecular separation, and trabecular bone volume/tissue volume, (Tb. Th) trabecular thickness. Data are presented as mean ± SD; ^#^ *P* < 0.01, *P < 0.05, **P < 0.01, ***P < 0.001.

To further demonstrate the potential dual therapeutic effects of velutin in the treatment of OA, we used histological staining and micro-CT to analyze cartilage degradation and SCB loss, respectively. First, Safranin O staining revealed that the AC of the experimental group (DMM) showed significant breakage and erosion of AC compared to the sham group (**Figure 2A**). Additionally, there was lower severity of destruction and degeneration of AC in the velutin-treated group than in the DMM group, and the color of Safranin O was much deeper. The treatment group’s OARSI scores were lower than those of the DMM group. (**Figure 2B**).

Next, we aimed to assess whether velutin has a protective impact on SCB loss. Through TRAP staining of SCB, a substantial increase in the number of TRAP (+) multinucleated cells in the DMM group was observed compared to the control group. We defined cells growing on the surface of the SCB trabeculae as Trap (+) cells. Velutin therapy, on the contrary, significantly reduced the number of TRAP (+) multinucleated cells. (**Figures 2C, D**). In addition, micro-CT images showed the same results; the DMM group showed considerable SCB loss compared to normal mice in the sham group. In contrast, intra-articular injection of velutin reduced SCB loss (**Figure 2E**). The ratio of bone volume to tissue volume (BV/TV), trabecular number (Tb. N), and trabecular spacing (Tb.Sp, mm) further confirmed that velutin suppressed osteoclast-mediated SCB loss (**Figures 2F–I**). Thus, velutin protected DMM-OA mice against AC degeneration as well as against osteoclast-mediated bone degradation *in vivo*.

### Protective effects of velutin against IL-1B-induced inflammation in chondrocytes and degradation of the ECM *in vitro*

**Figure 3:**
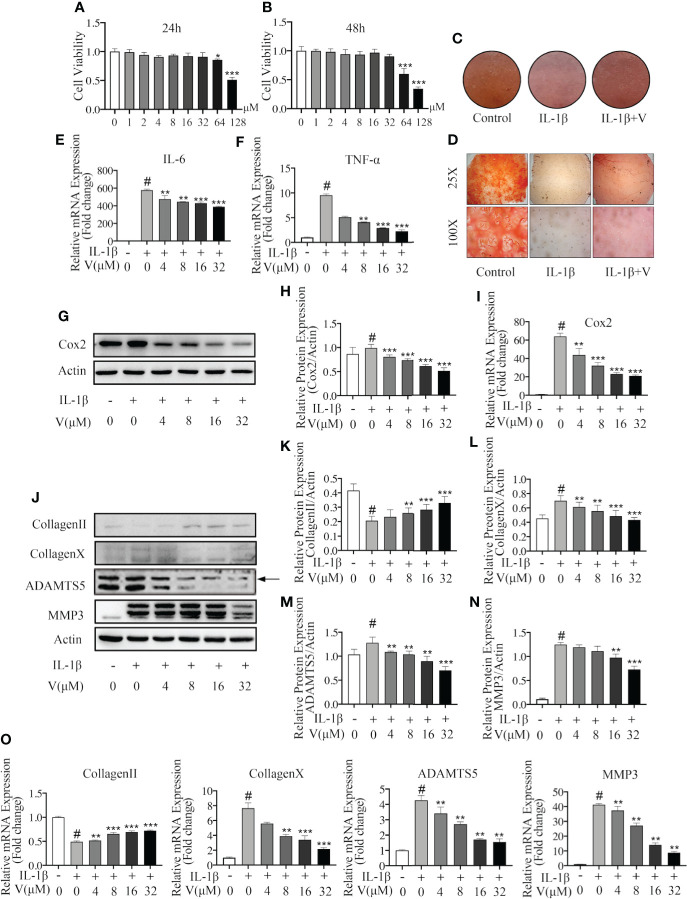
Velutin protects against IL-1β–induced chondrocyte inflammation and extracellular matrix degradation *in vitro*. **(A, B)** CCK-8 experiments revealing the cytotoxic effect of velutin on chondrocytes during 24 and 48 h at various doses (n = 5). **(C)** Safranin O staining in high-cell density culture of primary mouse chondrocytes. **(D)** Safranin O staining in human cartilage tissues. **(E, F)** qPCR of the mRNA expression levels of TNF-α and IL-6. **(G–I)** Velutin inhibited IL-1β-induced protein and mRNA expression of COX-2 in chondrocytes in a dose-dependent manner, according to Western blot and qPCR analysis. **(J–O)** Western blot and qPCR analyses showing that IL-1β treatment induced collagen II degradation and increased the expression of collagen X, MMP3, and ADAMTS5, whereas velutin treatment rescued these effects. Expression of target genes was normalized to β-actin and expressed as fold change in comparison to the controls group (n = 3). Data are presented as mean ± SD; ^#^ *P* < 0.01 vs. control group; *P < 0.05, **P < 0.01, ***P < 0.01 vs. IL-1β alone (n = 3).

Next, we evaluated the specific role of velutin in chondrocytes and osteoclasts *in vitro*. First, we assessed the cytotoxicity of velutin on chondrocytes by treating them with different concentrations of velutin (0, 1, 2, 4, 8, 16, 32, 64, and 128 μM) for 24 and 48 h. According to CCK8 results, there was no obvious change in the cytotoxicity from 0 to 32 μM (**Figures 3A, B**). Therefore, we used velutin doses of 4, 8, 16, and 32 μM in subsequent experiments.

Then, we tested the effect on the high cell density culture of primary mouse chondrocytes and human cartilage tissue samples using Safranin O and Fast Green staining. Compared to the control, the color intensity was lighter in the IL-1β-treated murine chondrocytes but darker in the velutin-treated chondrocytes, showing that velutin rescued the inflammatory response and protected against IL-1β-induced chondrocyte-mediated inflammation (**Figure 3C**). Similarly, IL-1β-treated human cartilage tissue samples showed a low color intensity of the Safranin O staining, more severe cartilage destruction, and amorphous morphology (**Figure 3D**). These effects were partially rescued by velutin treatment.

To further show that velutin exerts anti-inflammatory properties, we used IL-1β to treat chondrocytes at specific concentrations for 24 h, and RT-PCR and western blot experiments were conducted to determine the expression levels of RNA and proteins, respectively. RT-PCR showed that IL-1β treatment enhanced the mRNA expression levels of pro-inflammatory factors (TNF-α and IL-6), and velutin treatment significantly reduced mRNA expression levels compared to the sham group (**Figures 3E, F**). IL-1β treatment also promoted protein and mRNA expression of COX-2, whereas velutin treatment reversed this inflammatory response in a concentration-dependent way (**Figures 3G–I** ). In conclusion, these data confirm the dose-dependent, anti-inflammatory effects of velutin at the gene and protein levels.

Chondrocytes secrete the ECM (39); thus, we studied the effect of velutin in IL-1β-induced ECM degradation. We investigated the expression of type II collagen, type X collagen, MMP3, and ADAMTS5 by RT-qPCR and western blotting. Both RT-qPCR and western blotting results showed that IL-1β upregulated the expression of genes and protein representing ECM degradation (collagen X, ADAMTS4, and ADAMTS5). However, the velutin therapy group showed opposite consequences, which promoted the dose-dependent expression of genes and protein representing ECM synthesis (collagen II), as shown in **Figures 3J–O**. These results support that velutin is protective against IL-1β-induced ECM degradation and chondrocyte inflammation *in vitro*.

### Velutin attenuates RANKL-induced osteoclast formation and suppresses bone resorption *in vitro*

**Figure 4:**
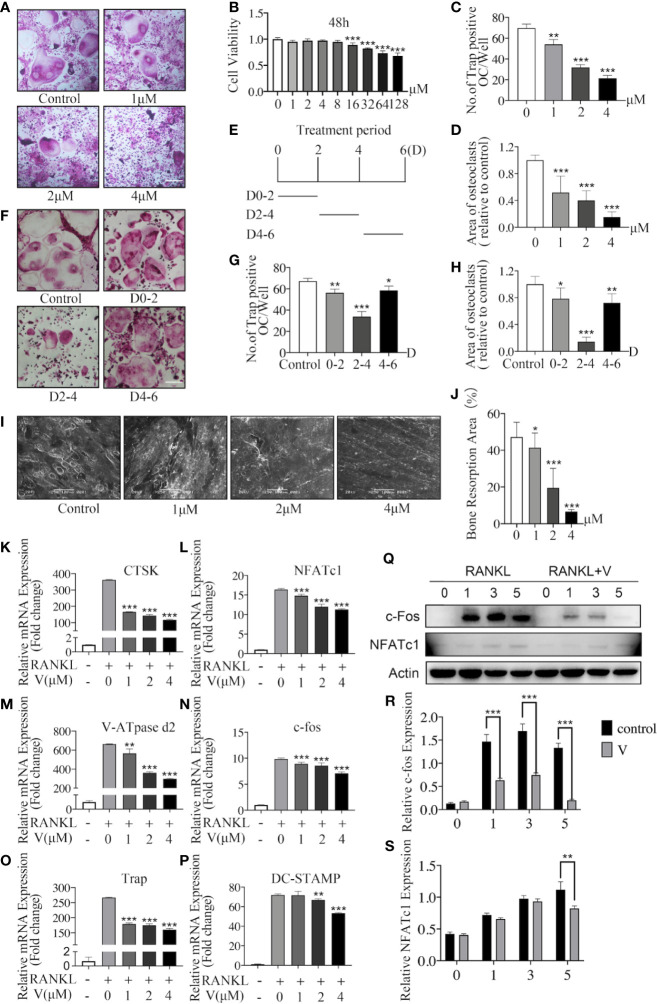
Velutin inhibits nuclear factor-κB receptor activator ligand (RANKL)-induced osteoclast formation and bone resorption *in vitro*. **(A)** Bone marrow-derived macrophages (BMMs) were grown for 6 days with M-CSF and RANKL, as well as varied velutin concentrations, before being stained for TRAP detection. Osteoclasts were defined as cells with three nuclei or less. (n = 3); scale bar: 200 μm. **(B)** CCK-8 assays showing the cytotoxicity of velutin in BMMs (n = 5). **(C-D)** Analysis of number and size (area) of TRAP‐positive multinucleated (nuclei > 3) cells (n = 3). **(E)** Schematic diagram of the timeline of BMM cells treated with velutin. **(F)** BMMs were cultivated in complete medium, then treated with velutin (4 μM) and stained for TRAP on the days indicated; scale bar: 200 μm. **(G-H)** Analysis of number and size (area) of TRAP‐positive multinucleated (nuclei > 3) cells (n = 3). **(I-J)** Bone tissue samples were used to cultivate M-CSF-dependent BMMs, then stimulated with RANKL and the specified velutin doses. SEM examination observed bone resorption lacunae after 15 days, which were calculated as percentages using Image J program (n = 3). **(K–P)** qPCR result of RANKL-induced osteoclast-related genes’ relative expression levels. Expression of target genes was adjusted to β-actin and expressed as fold change compared to the controls group (n = 3). **(Q–S)** M-CSF-dependent BMMs were serum-starved and pretreated for 2 h with velutin (4 μM) or vehicle control, before being stimulated with RANKL for the durations indicated times (0, 5, 15, 30, and 60 min). Western blot analysis was used to extract total cellular protein for protein expression levels (n = 3). Data are presented as mean ± SD; *P < 0.05, **P < 0.01, and ***P < 0.001, in comparison to cells treated only with RANKL.

To investigate the inhibition of velutin to osteoclast formation and differentiation *in vitro*, we initially looked at velutin cytotoxicity in BMMs by exposing cells to various velutin concentrations (0–128 μM) for 48 h. The viability of BMMs was unaffected by velutin at concentrations of 0 to 8 μM (**Figure 4B**). Therefore, velutin doses of 1, 2, and 4 μM were used in the following experiments.

Then, we used TRAP staining to visualize Trap activity and test the effects of velutin on osteoclastogenesis. As illustrated in **Figure 4A**, without velutin, RANKL stimulation induced the formation of osteoclasts, which are typically large and multinucleated. However, when cells were cultured with velutin, we found a dose-dependent decrease in cell size and the quantity of TRAP (+) osteoclasts. The number and area (size) of TRAP (+) multinucleated cells (cells with more than three nuclei) are displayed in **Figures 4C** **, D**.

Likewise, to determine the inhibitory effect of velutin on the development of osteoclasts, we cultured BMMs with velutin (4 μM) in a complete medium at three different periods (D0–2, D2–4, and D4–6; **Figure 4E**). The strongest inhibitory effect was observed on D2–D4, during which osteoclasts were significantly smaller and less multinucleated (**Figures 4F** **–H**). However, on D0–2 and D4–6, there was only a moderate reduction in osteoclast formation, indicating that velutin inhibits osteoclasts differentiation mainly in the mid-stage of the differentiation process. The above experimental results showed that exposure to velutin inhibited osteoclast development in a dose- and time-dependent manner *in vitro*.

Velutin inhibits RANKL-induced osteoclast formation by preventing precursor cell fusion. The next step was to see how velutin affected bone resorption in mature osteoclasts. BMMs were cultured with bone tissue samples after treatment with or without different concentrations of velutin for 15 days. In this study, we found a significant dose-dependent reduction of bone resorbed areas of mature osteoclasts after velutin treatment in comparison with untreated controls (**Figures 4I, J**).

Next, we investigated velutin effects on osteoclast formation and differentiation at gene and protein levels. BMMs were stimulated with RANKL, with or without velutin, for 1, 3, and 5 days, and the expressions of c-Fos and NFATc1 were evaluated. The expression of c-Fos and NFATc1 were robustly induced by RANKL (**Figures 4I** **, L**), whereas velutin treatment downregulated c-fos expression on days 1, 3, and 5. The inhibitory effect on NFATc1 expression was mainly observed on day 5 (**Figures 4Q** **–S**). As exhibited in **Figures 4K–P**, upon RANKL stimulation, osteoclast-related genes were upregulated to a different degree, whereas velutin reduced the mRNA levels of osteoclast-specific genes in a concentration-dependent manner. Our data showed that velutin prevented RANKL-induced mature osteoclast formation and impaired bone resorption *in vitro*.

### Velutin exerts a dual protective effect through inhibition of the p38 signaling pathway

The MAPK signaling pathway, which includes p38 MAPK, ERK, and JNK proteins, is partly responsible for cartilage destruction, osteoclast formation, and bone resorption in OA (40, 41).

**Figure 5:**
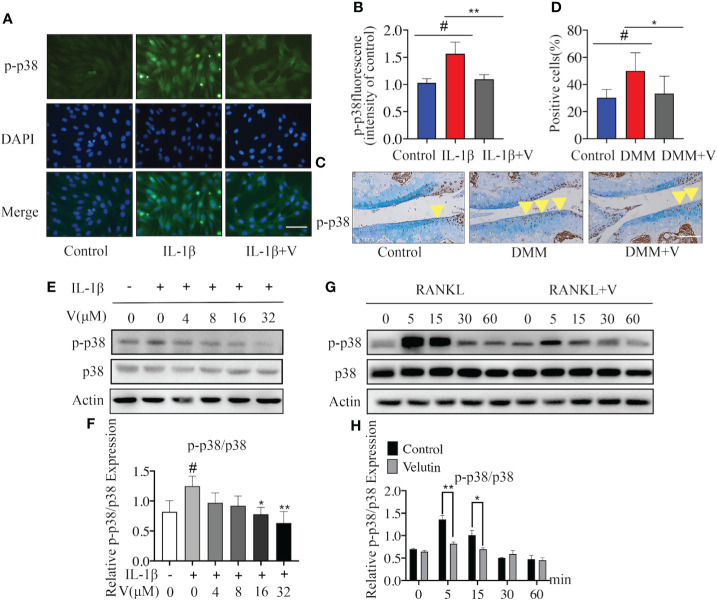
Velutin mediates its inhibitory effects through the p38 signaling pathway blockage. **(A, B)** Immunofluorescence DAPI nuclear staining showing that IL-1β promoted the expression of p-p38, velutin treatment reversed this effect. **(C, D)** Immunohistochemical staining of p-p38 MAPK in cartilage and percentages of p-p38 MAPK positive cells; the yellow triangle indicates positive cells. **(E, F)** Chondrocytes were pretreated with velutin (4 μM) for 2 h, then stimulated with or without IL-1β for 15 min. IL-1β promoted p38 phosphorylation, while velutin reduced it in a dose-dependent manner. The expression of p-p38 MAPK relative to total p38 MAPK was determined by ImageJ software. **(G, H)** M-CSF-dependent bone marrow-derived macrophages (BMMs) were serum-starved and given velutin (4 M) or a vehicle control for 2 h before being activated with nuclear factor-κB receptor activator ligand (RANKL) for the indicated times (0, 5, 15, 30, and 60 min). RANKL promoted p38 phosphorylation, whereas velutin reduced in a time-dependent way. ImageJ software was used to calculate the expression of p-p38 in comparison to total p38. Data are presented as mean ± SD; *P < 0.05, **P < 0.01, and ***P < 0.001, n = 3.

To assess the underlying mechanisms of the dual protective effects of velutin in a DMM mouse model, we first looked at how velutin affected IL-1-induced p38 signaling pathway initiation in chondrocytes. Immunofluorescence indicated that velutin expectedly decreased the expression of p-p38 MAPK (**Figures 5A, B**). Moreover, the immunohistochemical analysis also supported this finding, as the expression of p-p38 MAPK was dramatically lower in the velutin-treated group than that in the DMM group (**Figures 5C, D**). Furthermore, IL-1β dramatically increased the phosphorylation of p38 MAPK; however, this effect was dose-dependently reversed by velutin (**Figures 5E, F**). Additionally, the NF-κB signaling pathway is pivotal for inflammation in OA induced by IL-1β (42). Thus, we explored the effect of velutin on this pathway. Velutin treatment did not influence the phosphorylation of p65 as well as ERK and JNK (**Figures S1A, B**). These data show that velutin may mediate its effects in chondrocytes *via* the p38 signaling pathway.

Next, we studied the possible mechanism underlying the inhibition of osteoclast formation induced by velutin. We found that the activation and phosphorylation of p38 MAPK occurred rapidly and within 5 min following RANKL stimulation but then returned to basal levels after 15 min. In contrast, velutin treatment dramatically alleviated the phenomenon (**Figures 5G, H**). On top of that, velutin also reduced ERK and JNK phosphorylation (**Figures S1C, D**). Collectively, the protective effects of velutin against the deterioration of AC and the SCB loss are mediated *via* the inhibition of the p38 signaling pathway.

## Discussion

Osteoarthritis is a common chronic disease that causes functional decline and possible disability, thereby reducing the patients’ quality of life (43, 44). In the development and progression of osteoarthritis, a significant influence is played by the relationship between AC and SCB (45). In this study, we explored the effects of velutin extract in OA *in vivo* and *in vitro*. In an OA mouse model, we observed a protective effect of velutin against AC degradation and SCB loss. The DMM method was used to establish an OA model to test the protective effect of velutin in OA. DMM successfully induced OA in mice. In addition, in our study, DMM-induced OA mice treated with velutin showed reduced SCB loss and improved AC degradation, indicating that velutin exerted a dual protective effect against SCB remodeling and AC degeneration. These results were also confirmed *in vitro*.

Inflammation is significantly involved in OA progression (46, 47). Inflammatory mediators, especially IL-1β and TNF-α, are reportedly involved in OA progression, leading to the release of other pro-inflammatory cytokines such as COX-2 (48, 49). COX-2 is a crucial gene in OA development that causes inflammation and pain (49). In our study, velutin significantly downregulated the IL-1β-induced overexpression of COX-2 at the mRNA and protein level. In addition, MMPs regulate the cell-matrix composition and are involved in ECM degradation (50). MMP-3 is a key enzyme that modulates ECM degradation (51). ADAMTS enzymes, especially ADAMTS‐5, are primary aggrecanases that cleave aggrecans (52–54). Thus, we explored the effects of velutin on MMP3 and ADAMTS-5 and discovered that it protects against IL-1β-mediated MMP3 and ADAMTS-5 upregulation, suggesting that it may exert a protective role against ECM degradation induced by IL-1β.

Type II collagen and aggrecan are the main components of the ECM (50, 55); collagen X plays a role in ossification as markers of cartilage hypertrophy (56, 57). Collagen II expression is inhibited by IL-1β, thus resulting in the destruction of AC and facilitating the development of OA (58, 59). In addition, one of the important manifestations of OA is the degradation of aggrecan (54). Our study showed that velutin greatly suppressed the expression of collagen X while reversing the degradation of type II collagen, suggesting a protective effect against ECM degradation. Additionally, Safranin O staining of human cartilage tissue revealed that velutin significantly reduced the IL-1β-mediated inflammatory response. Immunohistochemical staining of aggrecan and type II collagen also demonstrated the protective effect of velutin in DMM-induced OA mice. Therefore, our data revealed a safeguarding effect of velutin against DMM-induced OA mice *in vivo* and IL-1β-mediated inflammation of chondrocyte and matrix degradation *in vitro*, providing evidence that velutin protects AC degradation during OA development. Previous studies have revealed that both the degeneration of AC and SCB loss are pathological characteristics in the early stage of OA (60). Targeting only cartilage degeneration without repairing the SCB loss would not effectively alleviate OA progression (61, 62). Therefore, it is an attractive therapeutic option for OA treatment to target SCB loss in OA.

Resorption of bone matrix and minerals and participation in the bone reconstruction cycle are the main functions of osteoclasts *in vivo* (63). M-CSF and RANKL, two key cytokines that induce the differentiation and fusion of OC precursor cells, are involved in a series of signaling events initiation that results in upregulating of genes related to osteoclast (22). Our results indicate that BMMs grown in media containing M-CSF and RANKL can develop into osteoclasts over time. Treatment with velutin, on the other hand, reduced the production of osteoclasts in a dosage and time-dependent way. This inhibitory effect was the most pronounced during the middle stage of osteoclast differentiation. In osteoclast formation, NFATc1 is the master transcriptional regulator, other genes including TRAP, c-Fos, CTSK, V-ATPase d2, and DC-STAMP are also important to OC formation and function (64). In this study, velutin significantly suppressed the protein and mRNA expression of above genes. Osteoclasts produce resorption pits by absorbing bone matrix and minerals leading to bone resorption (65). Therefore, we represent the formation of resorption pits as a proxy of osteoclast activity. We used bone slices as the mineral matrix to detect the resorption ability of seed osteoclasts *in vitro* and showed that velutin inhibited osteoclast formation and differentiation, which allowed us to further determine its protective effect on the SCB. Collectively, our results indicate that velutin had dual protective effects against AC degeneration and SCB loss.

The p38 signaling pathway exerts a crucial role in the pathophysiology of OA by increasing the production of inflammatory cytokines and ECM degradation products (66, 67). Inhibition of IL‐1β‐induced activation of p38 has been reported to attenuate OA progression (68). The phosphorylation of p38 MAPK, JNK, and ERK, which are MAPK family members, promotes osteoclast differentiation, initiating transcription of OC-related genes (69). In this study, velutin significantly inhibited the phosphorylation of p38 MAPK in chondrocytes and osteoclasts in a dose-dependent manner. Furthermore, immunofluorescence and immunohistochemistry analyses of p-p38 MAPK revealed that velutin significantly diminished IL-1β-induced upregulation of p-p38 MAPK. We hypothesized that the protective effect of velutin on AC degeneration and SCB loss is achieved through the p38 signaling pathway.

This study has certain limitations. We mainly focus on the inhibitory effects of velutin on OC formation and function but ignore the effect of OC fusion and migration, which are also necessary for osteoclast formation, which warrants further investigation. Moreover, we only used murine and not human chondrocytes. Although we found that velutin mediates its effects *via* the inhibition of the p38 signaling pathway, we have not identified a specific target for this effect. We also found that velutin inhibits osteoclasts through the ERK and JNK signaling pathways; however, it does not mediate its protective effect against chondrocyte inflammation through these signaling pathways. Therefore, we speculate that there are other targets for velutin to protect against cartilage degeneration and SCB loss, which requires further investigation.

In conclusion, our study revealed that velutin could protect against OA osteochondral pathologies and SCB loss caused by OA *in vivo*, impede the development of mature osteoclasts and bone resorption triggered by RANKL, and protect against IL-1β-induced chondrocyte inflammation *in vitro*. Velutin not only rescues AC degeneration but also prevents SCB loss, demonstrating its dual protective effect on DMM-induced OA in mice. In addition, we showed that velutin exerts its protective effects *via* the p38 signaling pathway; therefore, velutin might be a promising therapeutic target in the treatment of OA.

## Data Availability Statement

The original contributions presented in the study are included in the article/**Supplementary Material**. Further inquiries can be directed to the corresponding author.

## Ethics Statement

The studies involving human participants were reviewed and approved by Medical Ethics Committee of Shaoxing People’s Hospital, Shaoxing, Zhejiang Province, China. The patients/participants provided their written informed consent to participate in this study. The animal study was reviewed and approved by The Animal Ethical Committee of Shaoxing People’s Hospital, Shaoxing, Zhejiang Province, China.

## Author Contributions

KW, Investigation, Conceptualization, Validation, Writing - Original Draft, and Writing - Review and Editing. XLu, Conceptualization and Methodology. XLi, Resources. YZ, Investigation. RX, Software. YL, Visualization. YW, Methodology. TZ, Formal analysis. YQ, Methodology, Writing - Original Draft, Writing - Review and Editing. All authors contributed to the article and approved the submitted version.

## Funding

This work was supported by the National Natural Science Foundation of China [grant numbers 82072467 and 81871801], Natural Science Foundation of Zhejiang Province [grant number LQ21H060001], the Young Scientists Foundation of Shaoxing People’s Hospital [grant number 2021YA05] and Natural Science Foundation of Zhejiang Province [grant number LGF22H060031].

## Conflict of Interest

The authors declare that the research was conducted in the absence of any commercial or financial relationships that could be construed as a potential conflict of interest.

## Publisher’s Note

All claims expressed in this article are solely those of the authors and do not necessarily represent those of their affiliated organizations, or those of the publisher, the editors and the reviewers. Any product that may be evaluated in this article, or claim that may be made by its manufacturer, is not guaranteed or endorsed by the publisher.

## Abbreviations

OA: osteoarthritis; V: velutin; DMM: destabilization of the medial meniscus; BMMs: Bone marrow-derived macrophages; MMP: metalloproteinase; ECM: extracellular matrix; RANKL: receptor activator of nuclear factor-κ B ligand; M-CSF: macrophage colony-stimulating factor; DMSO: dimethyl sulfoxide; TRAP: tartrate‐resistant acid phosphatase; H&E: hematoxylin and eosin; CD: chondrocyte differentiation; IL-1β: interleukin-1β; IL-6: interleukin-6; TNF-α: tumor necrosis factor-α; AC: articular cartilage; CC: calcified cartilage; SCB: Subchondral bone.
