# Supplementary material for: Dual protective role of velutin against articular cartilage degeneration and subchondral bone loss via the p38 signaling pathway in murine osteoarthritis
Source: Front Endocrinol (Lausanne). 2022 Jul 22;13:926934. doi: 10.3389/fendo.2022.926934 (PMC9354239; doi:10.3389/fendo.2022.926934)
Supplement: Supplementary file 1 [file DataSheet_1.docx]

Supplementary Material

**
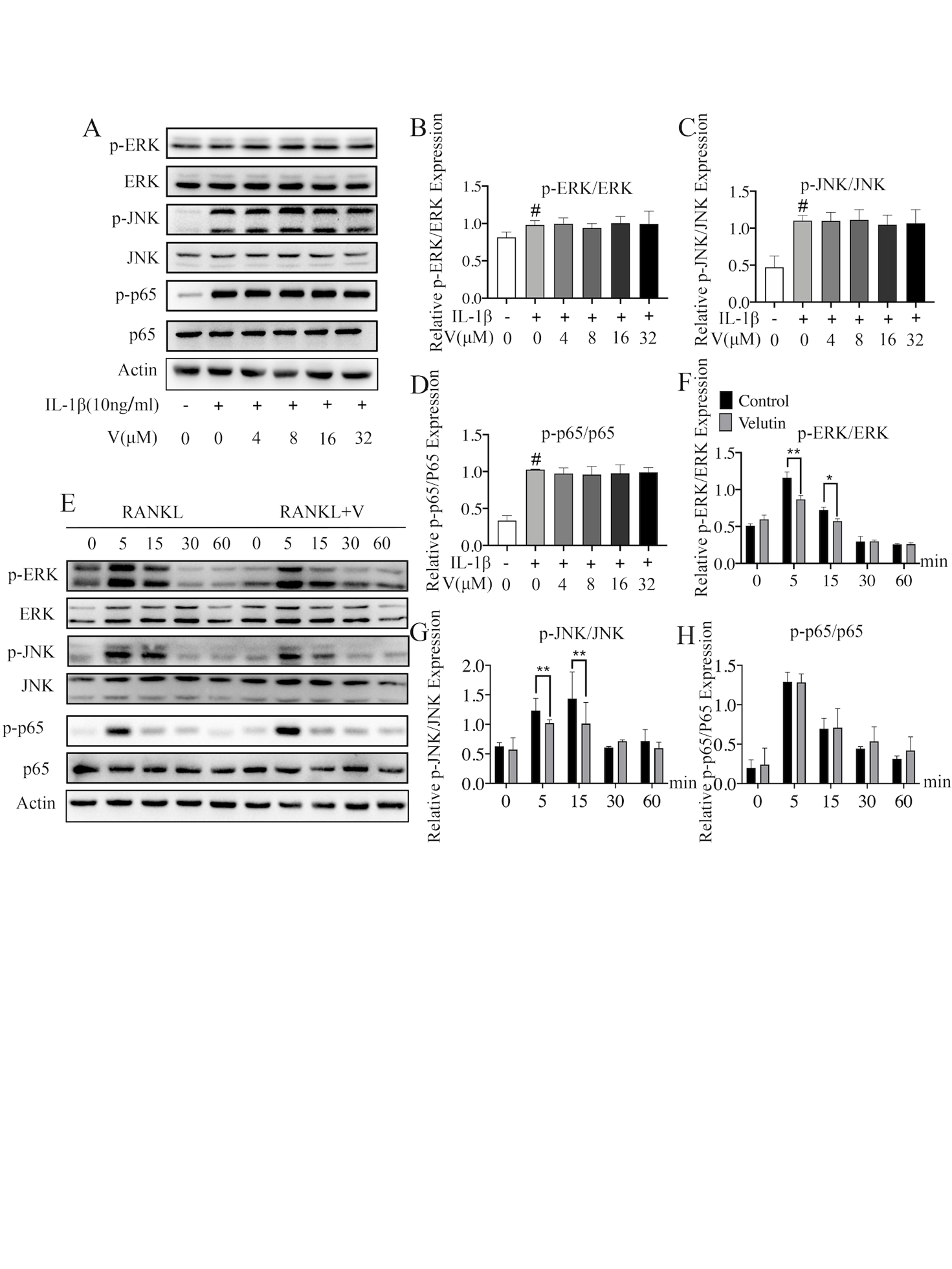
**

**Figure S1. Effects of velutin on MAPKs and NF-κB signaling pathways in mouse chondrocytes and BMMs. (A-C)** Chondrocytes were pretreated for 2 h with velutin (4 μM), followed by stimulation with or without IL-1β for 15 min. Image J software was used to measure the expression of p-ERK relative to total ERK, p-JNK relative to total JNK, and p-p65 relative to total p65. **(E-H**) M-CSF-dependent bone marrow-derived macrophages (BMMs) were serum-starved and pretreated with velutin (4 μM) or vehicle control for 2 h before being stimulated with nuclear factor-κB receptor activator ligand (RANKL) for the indicated time points (0, 5, 15, 30, and 60 min). Western blot analysis was used to extract total cellular protein to determine protein expression levels, (n = 3). The expression of p-ERK relative to total ERK, p-JNK relative to total JNK, and p-p65 relative to total p65 was determined using Image J software. Data are presented as mean ± SD; **P* < 0.05, ***P* < 0.01, and ****P* < 0.001, n = 3.
